# Uncertainty quantification patterns for multiscale models

**DOI:** 10.1098/rsta.2020.0072

**Published:** 2021-05-17

**Authors:** D. Ye, L. Veen, A. Nikishova, J. Lakhlili, W. Edeling, O. O. Luk, V. V. Krzhizhanovskaya, A. G. Hoekstra

**Affiliations:** ^1^ Computational Science Lab, Informatics Institute, Faculty of Science, University of Amsterdam, Amsterdam, The Netherlands; ^2^ Netherlands eScience Center, Amsterdam, The Netherlands; ^3^ Max Planck Institute for Plasma Physics, Garching, Germany; ^4^ Scientific Computing Group, Centrum Wiskunde and Informatica, Amsterdam, The Netherlands; ^5^ ITMO University, Saint Petersburg, Russia

**Keywords:** uncertainty quantification, uncertainty propagation, multiscale simulation, surrogate modelling

## Abstract

Uncertainty quantification (UQ) is a key component when using computational models that involve uncertainties, e.g. in decision-making scenarios. In this work, we present uncertainty quantification patterns (UQPs) that are designed to support the analysis of uncertainty in coupled multi-scale and multi-domain applications. UQPs provide the basic building blocks to create tailored UQ for multiscale models. The UQPs are implemented as generic templates, which can then be customized and aggregated to create a dedicated UQ procedure for multiscale applications. We present the implementation of the UQPs with multiscale coupling toolkit Multiscale Coupling Library and Environment 3. Potential speed-up for UQPs has been derived as well. As a proof of concept, two examples of multiscale applications using UQPs are presented.

This article is part of the theme issue ‘Reliability and reproducibility in computational science: implementing verification, validation and uncertainty quantification *in silico*’.

## 1. Introduction

Multiscale modelling and simulation has demonstrated its significance in computational sciences and engineering [1–3]. It allows scientists to study and simulate complex real-world phenomena at different scales and perspectives. A multiscale model usually consists of multiple single-scale submodels coupled by scale bridging methods.

Frequently, multiscale models include both epistemic uncertainty and aleatory uncertainty [4]. The former one is due to a lack of knowledge of the simulated system, e.g. uncertain input parameters, initial conditions, boundary conditions, etc. and the latter one refers to the natural stochasticity in the system. Therefore, uncertainty quantification (UQ) analysis is commonly needed. There are three main aspects of UQ: forward and inverse uncertainty propagation, and sensitivity analysis (SA) [5]. Uncertainty propagation deals with how the uncertainties propagate through the model and to the final output, quantifying the output uncertainty caused by the input and model uncertainties [6,7]. SA tackles the question of which uncertainties contribute most to the overall model output uncertainty, in the common situation of multiple uncertain sources [8,9]. The implementation of SA is similar to the forward propagation problem but with a more clever way to plan and generate samples. In this paper, we will mainly discuss the forward uncertainty propagation which is applicable to both UQ analysis and corresponding SA.

The UQ analysis of a multiscale simulation is usually implemented using a non-intrusive method such as a Monte Carlo method [10], polynomial chaos expansion (PCE) [11], or surrogate modelling techniques [12,13]. By exploiting the computational structure of multiscale simulation, the cost of a multiscale UQ can be reduced by relying on a family of recently proposed semi-intrusive UQ methods [14]. In this manuscript, we extend the notion of exploiting generic computational structure of multiscale simulations to further improve the computational efficiency by identifying generic UQPs, and then implementing the UQ analysis with these UQPs. In fact, we propose a series of uncertainty quantification patterns (UQPs) according to the degree of intrusion and architecture of multiscale simulation. These UQPs provide basic building blocks for creating tailored UQ for multiscale models. The UQPs are implemented as generic templates, which can then be customized and aggregated to create a dedicated UQ procedure for multiscale applications.

The paper is arranged as follows. Multiscale model and simulation framework (MMSF), uncertainty propagation and semi-intrusive UQ are introduced in §2 as they provide the basis upon which the UQPs are built. Section 3 characterizes the UQPs and their corresponding optimization patterns. Section 4 describes the implementation of the UQPs with the MMSF-based coupling toolkit MUSCLE 3 [15]. Applications scenarios from the field of plasma fusion physics and reaction–diffusion models are presented in §6.

## 2. Background

### (a) Multiscale modelling and simulation framework

The MMSF is a theoretical and practical framework to model, characterize and simulate multiscale phenomena [16,17]. It provides an abstract way to understand the (computational) structure of multiscale simulations. Without going into detail here, we will highlight a few notions from the MMSF that will guide the definitions and design of UQPs. In a multiscale model, two or more processes take place on one or more spatio-temporal domains at different scales. For each pair of processes, the relative scale and position of their domains determine the required coupling between the submodels representing them. An acyclic coupling is used if one process occurs prior to another, or can be modelled as such. In this case, one single-scale model provides input to the other, and each single-scale model is executed once. A cyclic coupling occurs if the processes occur at the same time and are time-scale separated. In this case, the slow dynamics (macro) model calls the fast dynamics (micro) model in an iterative loop, thus executing it many times.

Information flows through an acyclic model from the inputs and parameters, through the submodels and the couplings between them, to the quantities of interest (QoI) produced. This forms a directed acyclic graph, so that it is possible (although uncommon) for a result to depend on the same input or parameter via two different dependency paths. However, information will never feed back into a model that produced an output it depends on. In a cyclic model, information flows back and forth between two or more submodels, from the macromodel state at time step *t* through the micromodel and then back to the macromodel, where it informs its state at time step *t* + 1. The macromodel state at *t* + 1 typically depends on both the state at *t* and on the input from the micromodel (which in turn depends on the macromodel state at *t* as well), so that for cyclic models, multiple paths to the same dependency are usually present. (They may also occur if the micromodel saves its state in between runs.)

### (b) Uncertainty propagation

In a forward UQ problem, the information propagating through the model consists of uncertainty. When we deal with propagation of uncertainty in a multiscale simulation, the parameters, inputs, and information passed between models and the QoIs are random variables. Each model output depends on the model inputs and the parameters used, and thus the output random variable is conditional on these dependencies. If two inputs of a submodel have a shared dependency (e.g. they were produced by two different models which share an uncertain input or parameter), then the corresponding random variables may be correlated. As described above, this is rare in acyclic models, but usually the case in cyclic models. To obtain correct results, care must be taken to preserve this correlation. For instance, this may prohibit resampling one of the inputs independently of the other.

### (c) Semi-intrusive uncertainty quantification

A family of semi-intrusive algorithms for multiscale simulation UQ [14] has been proposed, which provide accurate estimates of output uncertainties at a significantly reduced computational cost compared to non-intrusive methods. The methods are semi-intrusive in the sense that they are intrusive only on the level of the multiscale model, but the single-scale components are viewed as black-boxes. There are two algorithms proposed for semi-intrusive UQ.

The first algorithm is the semi-intrusive Monte Carlo (SIMC) method. Instead of calling and running the computationally most expensive microscale submodel repeatedly, an interpolation based on a few calculations of the original model is used to reduce the computational cost. For instance, at time step *t*, the macro model passes a sample of size *n* of output ${\left\{u_{i}^{t}\right\}}_{i=1}^{n}$ to the micro model and asks for a corresponding response ${\left\{v_{i}^{t}\right\}}_{i=1}^{n}$. The number of executions of the micro model is significantly reduced if an interpolation based on ${\left\{v_{i}^{t}\right\}}_{i=1}^{\hat{n}}$, where $\hat{n}\ll n$, is applied to achieve the rest of the responses ${\left\{v_{i}^{t}\right\}}_{i=\hat{n}+1}^{n}$. The exact interpolation method can be selected depending on the particular model. At the same time, a validation using the training dataset ${\left\{v_{i}^{t}\right\}}_{i=1}^{\hat{n}}$ is carried out to estimate how much the UQ estimation would be affected by the interpolation error. It is quantified by $\varepsilon_{E}=|E[u^{t+1}]-E[{\hat{u}}^{t+1}]|$ and $\varepsilon_{\sigma }=|\sigma [u^{t+1}]-\sigma [{\hat{u}}^{t+1}]|$ statistically, where ${\hat{u}}^{t+1}$ is the macro output based on the interpolation at the next time step. If they are larger than a set threshold, the training sample size can be increased or another approximation method can be tested. The second algorithm is semi-intrusive metamodelling, where the most computationally intensive submodel is replaced by a surrogate model, which approximates the model output with a relatively low computational cost.

The design of UQPs is mainly inspired by the semi-intrusive concept, where the coupled structure of the multiscale model is explored. And the semi-intrusive algorithms mentioned above are the prototypes of some of the UQPs (i.e. from SIMC to UQP3-A, from semi-intrusive metamodeling to UQP2/3-B). However, it is important to note that the implementation of UQP is not limited to a specific method. For example, although we designed UQP3-A with the semi-intrusive ‘Monte Carlo’ algorithm, the interpolation-based method mentioned above can also be applied to other UQ methods, such as PCE (non-intrusive) [11] or stochastic collocation [11] in which a number of simulations have to be carried out for UQ analysis.

## 3. Uncertainty quantification patterns

**Figure 1.**
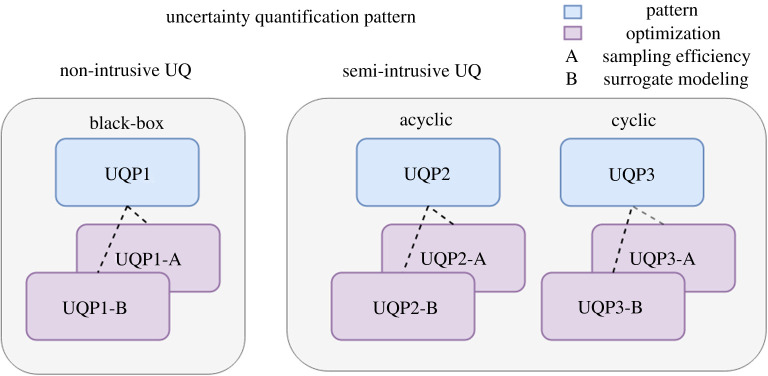
The summary of uncertainty quantification patterns. UQP1 is taken as a black-box pattern where the general non-intrusive UQ methods can be applied. UQP2 and UQP3 are based on the semi-intrusive UQ methods which exploit the coupling in multiscale simulations to improve the efficiency of UQ. Each pattern can further improve the computational efficiency by applying efficient sampling (A), or surrogate modelling techniques (B). (Online version in colour.)

In this section, we characterize the details of UQPs and the corresponding optimization methods. An overview of the UQPs is shown in figure 1. We categorize the UQPs according to the degree of intrusion and architecture of multiscale simulation. The first category, UQP1, is a pattern which represents the commonly used non-intrusive methods. UQP2 and UQP3 are based on semi-intrusive methods and apply to acyclic and cyclic multiscale models, respectively. In addition, we consider two ways to optimize the computational efficiency: efficient sampling (A) and surrogate modelling (B). Efficient sampling refers to sampling techniques more efficient than basic methods and hence reduces the number of samples required to perform UQ for the given set of input parameters. Surrogate modelling refers to replacing the computationally expensive model or submodel by a surrogate. The surrogate model approximates the behaviour of the original model at a lower computational cost.

### (a) UQP1: Non-intrusive pattern

**Figure 2.**
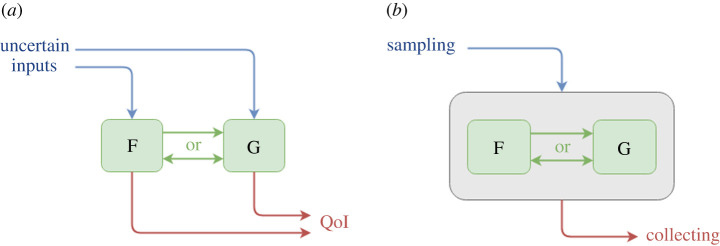
(*a*) Prototypical multiscale model. F and G are two submodels of a multiscale simulation coupled in a general sense. Both submodels take uncertain inputs and output QoIs with uncertainties (*b*) UQP1 considers the multiscale model as a black-box that has inputs and produces outputs. (Online version in colour.)

Consider a prototypical multiscale model consisting of two submodels, F and G, coupled together in the most general sense, either acyclic or cyclic, as shown in figure 2*a* by the arrows in between submodels. Both submodels F and G take uncertain inputs, e.g. initial conditions, boundary conditions or model parameters (shown by blue incoming arrows) and both produce QoIs with uncertainties (the red outgoing arrows). The simplest UQP, called UQP1, does not exploit the multiscale simulation structure, and considers the multiscale model as a black box that has inputs and produces outputs. As shown in figure 2*b*, the UQ is performed by using non-intrusive methods on the application as a whole, and quantifying the uncertainty relative to a QoI that is part of the final application output.

To optimize the computational efficiency of UQP1, one can apply advanced sampling methods to reduce the total number of runs of the simulation (UQP1-A). In addition, a low-cost surrogate model can be built based on the mapping between uncertain inputs and the QoI to replace the model as a whole (UQP1-B). Although this is the most common way to implement the UQ analysis for a computational model, the method can be computationally prohibitive for many multiscale applications that require significant computational resources. Therefore, in the next section, we discuss how the multiscale structure of such applications can be exploited in order to perform uncertainty propagation more efficiently.

### (b) UQP2: Semi-intrusive acyclic pattern

**Figure 3.**
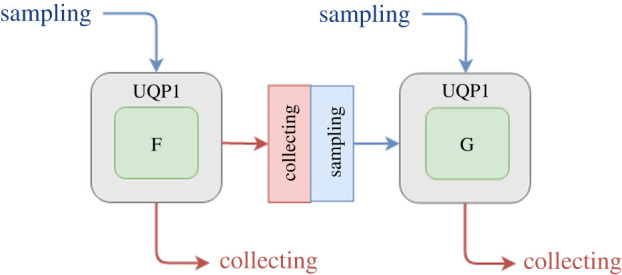
UQP2: Semi-intrusive acyclic pattern. UQP2 performs non-intrusive UQ on submodels. After applying UQP1 to submodel F, the data to be sent to submodel G has now turned into an uncertain output which then is converted into uncertain input for submodel G. (Online version in colour.)

According to the coupling topology of the multiscale simulation, a main distinction can be made between acyclic and cyclic multiscale models. In the case of acyclic structure, uncertainty propagates in one direction through the multiscale model. Output uncertainty of one single component creates input uncertainty of another component. UQP2 performs non-intrusive UQ on consecutive single components in an acyclic model (figure 3).

There are two important advantages of applying UQP2: transparency and efficiency. UQP2 makes it possible to investigate how uncertainty propagates and amplifies within each component of the model. UQP2 can be realized as a sequential application of UQP1 (figure 3). After applying UQP1 to submodel F, the data to be sent to submodel G has now turned into an uncertain output, which is then converted into uncertain input for submodel G. Therefore, UQP2 makes uncertainty propagation more transparent and provides additional information on uncertainty as it propagates between submodel levels.

The second important advantage of UQP2 is that it introduces different ways of improving the computational efficiency of corresponding UQ analysis. One way to obtain better efficiency is to apply resampling: samples of the output of one submodel can be used to approximate the probability density function (PDF) of this output. Then, this output can be considered along with other uncertain inputs of the next submodels and, therefore, uncertainty propagation of this submodel can be performed independently of the analysis of the previous one. This allows applying the most efficient uncertainty propagation methods for each of the single-scale models. This also allows for a more flexible implementation of UQP2-A, in which sampling for each submodel can apply a different advanced sampling method. Another way to improve the efficiency for acyclic multiscale models is to build a metamodel of the most expensive single-scale model.

### (c) UQP3: Semi-intrusive cyclic pattern

**Figure 4.**
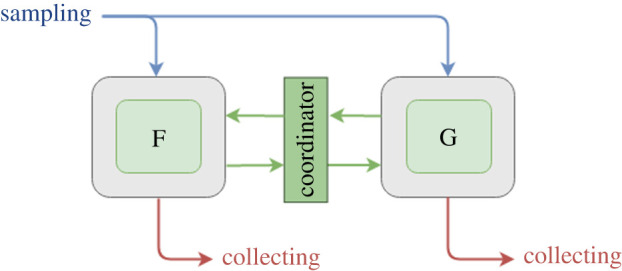
UQP3: Semi-intrusive cyclic pattern. UQP3 again performs non-intrusively on consecutive single components. Compared to UQP2, UQP3 has an additional box (Coordinator) between the submodels, to orchestrate the subsampling, interpolation and statistical testing of the interpolation. (Online version in colour.)

For cyclic multiscale models, we propose UQP3 as shown in figure 4. UQP3 again performs non-intrusive UQ on individual single components. Compared to the UQP2, in UQP3 we add the Coordinator module between the submodels, to orchestrate the data flows. For UQP3, resampling cannot be applied as it can be for acyclic UQP2 workflows, because the models alternate to modify the model’s state, which causes their states and parameters to become correlated as we explained in §2b. The output of the first model must be sampled conditionally on the value of the shared parameters for the target second model instance. Therefore, instead of resampling, the SIMC method (explained in §2c) can be applied to improve the efficiency (UQP3-A). Using the Coordinator between the submodels, one can deploy the corresponding interpolation and the statistical validation. For cyclic multiscale models, the states of the submodels become correlated even if their parameters are separated, through the repeated communication between the submodels. UQP3-A can be applied in this situation as well. Additionally, it is important to note that the implementation of UQP3-A is not limited to the Monte Carlo methods. Other UQ methods, such as PCE or stochastic collocation, can be applied to reduce its computational cost and improve the efficiency of the multiscale UQ analysis.

For UQP3-B, again each submodel can be replaced by a surrogate model that produces an approximation significantly faster than the original submodel. In a time-scale separated coupling, the micro model will run many more time steps than the macro model, so it is usually the target for surrogate modelling. A distinction can be made between stateful and stateless submodels. In a stateful case, the output of the mircomodel depends on all previous inputs. Timescale overlapping couplings are similar to timescale separated couplings with a stateful micro model, but will not be discussed further here.

Besides, the Coordinator in UQP3 allows us to implement UQ with an online surrogate model. Instead of deploying a pre-trained surrogate model, one can dynamically and adaptively train and test the surrogate model during the UQ implementation. Such a process is similar to the concept of active learning [18,19], but embedded in a UQ procedure. This concept of online surrogate model can be an interesting research topic to further improve the computational efficiency of UQ.

## 4. Implementing uncertainty quantification patterns using Multiscale Coupling Library and Environment 3

In this section, we describe how the UQPs can be implemented using the Multiscale Coupling Library and Environment (MUSCLE 3) [15]. MUSCLE 3 is a coupling framework that is designed for coupling temporally and/or spatially scale-separated multiscale models. In a MUSCLE 3 simulation, submodels run simultaneously as separate programs and exchange information via the network, through the libmuscle library. Each submodel has one or more *ports*, which are named connectors through which it sends and receives messages. The ports of the submodels are connected by MUSCLE according to a description in a configuration file based on an extended version of the Multiscale Modelling Language [16]. As a result, substituting one submodel for another or adding in generic components that help implement the UQPs is as simple as changing the configuration file. Different configurations of the model can therefore be tracked simply by storing a file for each, which is much easier than having to manage multiple parallel versions of the submodels’ source codes. Here, we will use a graphical representation of MML to depict the required couplings.

The configuration file also contains model settings (parameters and other configuration), and MUSCLE 3 has a mechanism through which one model component can send new settings to another component. This settings overlay overrides global settings, and is automatically propagated to subsequent connected components. Many copies of a (sub)model can be instantiated, which combined with the settings overlay allows for implementing UQ, as described below.

### (a) UQP1: Non-intrusive pattern

**Figure 5.**
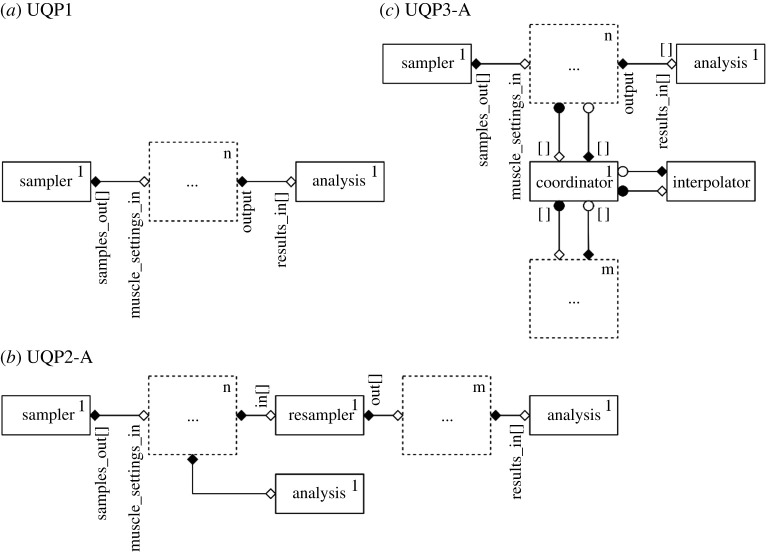
Implementing UQPs using MML and MUSCLE 3: (*a*) UQP1, (*b*) UQP2-A and (*c*) UQP3-A. Boxes represent components with their number of instances in the top-right corner, dashed boxes are placeholders for submodels, which are substituted into the pattern. Lines denote conduits through which data may be sent. An open diamond receives initialization data, a closed diamond sends the final result, a closed circle sends an intermediate output at each time step, and an open circle receives state or boundary conditions at each time step.

The UQPs are flexible with respect to which specific UQ methods are used. In this section, we use plain Monte Carlo as an example, as it is simple and well known. The Monte Carlo method for performing a forward UQ comprises three steps: (i) sampling the uncertain parameters *n* times, (ii) running the model once for each sampled value and (iii) calculating statistics of the resulting set of the outputs. For a multiscale model coupled using MUSCLE 3, this can be implemented by adding two additional components, a *Sampler* which samples the uncertain parameters, and an *Analysis* component, which calculates statistics, and connecting them to *n* instances of the original model (figure 5*a*). The Sampler produces *n* samples and sends them to its samples_out port. This is a vector port (shown by the square brackets), which is used to connect to a set of submodel instances, rather than to a single instance. It is connected to the muscle_settings_in scalar port of the existing model. This port implements the settings overlay mechanism described above, so that each instance of the original model will run with the corresponding parameters. The output of the model runs is then sent to the Analysis component, which receives both the results and the parameter overlay for each instance, which gives it all the information it needs to calculate the required statistics.

In order to implement UQP1-B, a surrogate model needs to be constructed, and substituted for the original model. This can be done by changing the configuration file to wire in the new component instead of the original model.

### (b) UQP2: Semi-intrusive acyclic pattern

In UQP2, we apply UQP1 to each of two models coupled sequentially (figure 5*b*). The *Sampler* and *Analysis* components of UQP1 are reused here. Alternatively, two separate Analysis modules can be used, one for each submodel. In this case, only marginal distributions and correlations among each submodel’s QoIs can be estimated, but not correlations between the different submodels’ QoIs. The submodels remain unchanged, as in UQP1.

To allow the use of different ensemble sizes or UQ methods for the two submodels (UQP2-A), a *Resampler* component is added in between. It receives *n* outputs of the first submodel on its vector port in, and converts those into *m* inputs for the second submodel, which it sends on its vector port out. Like the Analysis component, the *Resampler* receives both the first submodel’s output and its settings overlay. It is used to produce a new set of settings and corresponding inputs, and sends those on to the second submodel. Using surrogate models for individual submodels (UQP2-B) can be done just as for UQP1 by changing the configuration to substitute a surrogate for the component.

### (c) UQP3: Semi-intrusive cyclic pattern

Cyclic coupled models of time-scale separated processes consist of a macro model that at each time step calls a micromodel, which runs to convergence and sends a result back to the macro model. To implement UQP3, we can first apply UQP1 by connecting the *Sampler* and *Analysis* components to the macro model, and instantiating *n* copies of both submodels (figure 5*c*). Here too parameters will be automatically propagated to the micromodel, and both submodels remain unchanged.

For UQP3-A, the SIMC method explained in §2c is applied. The algorithm is generic, but the specific interpolation function used is model-specific. We therefore refine the scheme from §3 slightly, adding the generic *Coordinator* component, which implements the generic algorithm, but attaching to it a model-specific *Interpolator*, which interpolates the micromodel output in a model-specific manner (figure 5*c*).

The *Coordinator* component, being wired in between the two sets of submodel instances, intercepts messages from each ensemble member, including the parameters for that ensemble member. Messages from the initial *m* macro model instances are sent on to their corresponding micromodel instances, and the replies are then passed back. Thus, these ensemble members run normally. However, the micromodel outputs and their corresponding parameter settings are also sent to the Interpolator, which stores them. The remaining *n* − *m* messages and parameters are not forwarded to the micromodel, but instead the parameters are sent by the Coordinator to the Interpolator, which interpolates the previously stored messages to produce estimates of the micromodel output for the remaining *n* − *m* parameters. It sends these back to the Coordinator, which forwards them on to the corresponding macro model instances. Thus, the remaining *n* − *m* ensemble members use interpolated values, saving this many runs of the micromodel. Once micromodel results have been sent for all ensemble members, the macro model proceeds to its next time step. A more advanced version of the algorithm adds a validation step and dynamically adds more actual micromodel runs until the Interpolator returns results of sufficient quality.

For UQP3-B, a (pre-trained) surrogate can be substituted for the micromodel by changing the configuration. Training a surrogate while running can be done in a similar manner, substituting a surrogate model for the Interpolator. This requires the Coordinator to work slightly differently, sending micromodel input/output pairs, rather than parameters/output pairs. If the micromodel is stateful, then its output at time *t* depends on all messages sent to it at previous simulation time steps. In this case, the messages must be accumulated either by the Coordinator or by the surrogate model to produce a good prediction.

## 5. Speed-up of uncertainty quantification patterns

The motivation to perform UQ with one of the proposed UQPs is the speed-up that can be obtained by exploiting the multiscale structure of multiscale models. Hence, here we estimate possible gains in the computational time from the proposed algorithms.

Practical multiscale models often exhibit a dramatic difference between the computational cost of the single-scale models. Here, for the sake of simplicity, we consider a case of a multiscale model with two submodels: a computationally cheap macro model *M* and an expensive micro model *μ*. In this case, the computational cost of UQ will come mostly from the uncertainty propagation through the computationally expensive micro model. Therefore, below we discuss how the cost of UQ at the micro level is decreased when the proposed UQPs are applied.

### (a) Acyclic coupled models

#### (i) UQP1

Let us consider an uncertainty estimation method, where the number of samples grows exponentially with the number of uncertain inputs. An example of such a method may be a quadrature rule. Let us denote by *n* the number of samples for each uncertain input parameter, and by *d*_*M*_ and *d*_*μ*_, the number of uncertain inputs for the macro and micro models, respectively. Then, the computational cost of UQ stemming from the micro model in UQP1 is given by

5.1 $$C_{{\text{UQP1}}_{\mu }}=n^{d_{M}+d_{\mu }}C_{\mu },$$

where *C*_*μ*_ is the computational cost of one execution of the micro model.

#### (ii) UQP2-A

First, let us consider an example where the micro model is run first in an acyclic multiscale model. When UQP2 is applied to this example, the UQ cost for the micro model is

5.2 $$C_{{\text{UQP2-A}}_{\mu }}=n^{d_{\mu }}C_{\mu }$$

and the speed-up of the semi-intrusive approach UQP2 over the non-intrusive UQP1 is

5.3 $$\frac{C_{{\text{UQP1}}_{\mu }}}{C_{{\text{UQP2-A}}_{\mu }}}=\frac{n^{d_{M}+d_{\mu }}C_{\mu }}{n^{d_{\mu }}C_{\mu }}=n^{d_{M}}.$$

Alternatively, when the micro model is run after the macro model, the UQ cost on the micro scale is

5.4 $$C_{{\text{UQP2-A}}_{\mu }}=n^{d_{\mu }+d_{y}}C_{\mu },$$

where *d*_*y*_ is the dimensionality of the macro model output. In this case, the speed-up is

5.5 $$\frac{C_{{\text{UQP1}}_{\mu }}}{C_{{\text{UQP2-A}}_{\mu }}}=\frac{n^{d_{M}+d_{\mu }}C_{\mu }}{n^{d_{\mu }+d_{y}}C_{\mu }}=n^{d_{M}-d_{y}}.$$

Therefore, a speed-up is obtained if *d*_*M*_ > *d*_*y*_.

#### (iii) UQP2-B

In the semi-intrusive acyclic UQP2-B algorithm, the expensive micro model is replaced by a surrogate and then a standard method, like the Monte Carlo, can be applied for UQ. Therefore, here we assume that *N* is the total number of multiscale model runs (which may or may not depend on the number of uncertain inputs), and $\tilde{N}$ is the total number of expensive micro model runs such that $\tilde{N}\ll N$. The rest of the samples ($N-\tilde{N}$) are obtained using a surrogate that produces an approximation of the micro model results, but in a significantly shorter computational time

5.6 $$C_{{\text{UQP2-B}}_{\mu }}=\tilde{N}C_{\mu }+(N-\tilde{N})C_{\mu }^{*},$$

where $C_{\mu }^{*}$ is the computational cost of receiving one prediction from the surrogate. Then, the speed-up is

5.7 $$\frac{C_{{\text{UQP1}}_{\mu }}}{C_{{\text{UQP2-B}}_{\mu }}}=\frac{NC_{\mu }}{\tilde{N}C_{\mu }+(N-\tilde{N})C_{\mu }^{*}},$$

where a speed-up is achieved when $C_{\mu }^{*}<C_{\mu }$.

### (b) Cyclic coupled models

#### (i) UQP1

In the cyclic case, the cost of UQ from the micro model is the number of samples *N* required to obtain a reliable estimation of uncertainty, multiplied by the total number of times the micro model is called per simulation $N_{\Delta t_{M}}$

5.8 $$C_{{\text{UQP1}}_{\mu }}=N_{\Delta {\text{t}}_{M}}NC_{\mu }.$$

#### (ii) UQP3-A

The idea of UQP3-A is to run the expensive micro model a significantly lower number of times, i.e. *N*_*μ*_ ≪ *N*. The rest of the samples of the micro model response are obtained applying the interpolation with these *N*_*μ*_ samples used as a training set. Therefore, the total computational cost of UQ on the micro level is

5.9 $$C_{{\text{UQP3-A}}_{\mu }}=N_{\Delta {\text{t}}_{M}}(N_{\mu }C_{\mu }+(N-N_{\mu })C_{\mu }^{*}),$$

where $C_{\mu }^{*}$ is the cost to obtain one sample using the interpolation. The speed-up is then

5.10 $$\frac{C_{{\text{UQP1}}_{\mu }}}{C_{{\text{UQP3-A}}_{\mu }}}=\frac{N_{\Delta {\text{t}}_{M}}NC_{\mu }}{N_{\Delta {\text{t}}_{M}}(N_{\mu }C_{\mu }+(N-N_{\mu })C_{\mu }^{*})}=\frac{1}{\left(\frac{N_{\mu }}{N}+(1-\frac{N_{\mu }}{N})\frac{C_{\mu }^{*}}{C_{\mu }}\right)}.$$

Hence, if *N*_*μ*_ ≪ *N* and the obtained interpolation produces the micro model approximation much faster than the original micro model, i.e. $C_{\mu }^{*}\ll C_{\mu }$, then a significant speed-up is obtained.

#### (iii) UQP3-B

In UQP3-B, instead of the expensive micro model, a pretrained surrogate is used that requires $C_{\mu }^{*}$ to execute. Therefore,

5.11 $$C_{{\text{UQP3-B}}_{\mu }}=N_{\Delta {\text{t}}_{M}}NC_{\mu }^{*}+C_{k},$$

where *C*_*k*_ is the cost of constructing the surrogate, for instance, sampling and training. This would provide the speed-up

5.12 $$\frac{C_{{\text{UQP1}}_{\mu }}}{C_{{\text{UQP3-B}}_{\mu }}}=\frac{N_{\Delta {\text{t}}_{M}}NC_{\mu }}{N_{\Delta {\text{t}}_{M}}NC_{\mu }^{*}+C_{k}}=\frac{C_{\mu }}{C_{\mu }^{*}+\frac{1}{N_{\Delta {\text{t}}_{M}}N}C_{k}}.$$

Hence, as it is for the previous UQP, the cost of the surrogate $C_{\mu }^{*}$ together with the one to obtain it will define the magnitude of the speed-up.

## 6. Case studies

In this section, we present two case studies, acyclic and cyclic, that illustrate the efficiency gained when UQ is performed according to the UQPs.

### (a) Case study one: acyclic multiscale application

To demonstrate the acyclic multiscale case, we present a plasma fusion physics model comprising two single-scale deterministic models: (i) a transport solver, which evolves temperature profiles of the plasma at the macro time scale, and (ii) a 2D Equilibrium model, which updates the plasma geometry [20]. These two submodels are performed using serial codes that take respectively 10 ms and 1–5 s per run. Thus the equilibrium model is much more expensive to run computationally. In the previous work, we have carried out the uncertainty quantification with non-intrusive methods (UQP1) like the quasi-Monte Carlo method and PCE [21,22]. With a small number of uncertain parameters we can run each code with a low number of samples. To further improve the computational efficiency of the UQ, here we use UQP2 by applying UQP1 to each submodel with PCE and exploit the resampling in between submodels to achieve extra speed-up.

We consider six uncertain parameters coming from external heating sources and boundary conditions (4096 samples using PCE with a cubic polynomial [21]). The first UQP1 box (1D transport model) calculates the distribution of electron temperatures *T*_*e*_ as an output. The second UQP1 box (2D equilibrium model) takes *T*_*e*_ as input and calculates the plasma pressure *P*. In this case, the quantity *T*_*e*_ is a smooth profile evaluated across the radial grid coordinates *ρ*_tor_. Thus, the output of the first UQP1 box is a very high-dimensional, strongly (serially) correlated distribution. In order to reduce both correlation and dimensionality, we approximate each *T*_*e*_ sample with a cubic BSpline [23]:

6.1 $$T_{e}(\rho_{\text{tor}})=\sum_{i=1}^{4}C_{i}P_{i}(\rho_{\text{tor}}),$$

where *P*_*i*_ is a piece-wise polynomial of degree 3 and *C*_*i*_ are the spline coefficients. We then resample *C*_*i*_, allowing us to perform UQ for the equilibrium with less cost and sufficient accuracy.

**Figure 6.**
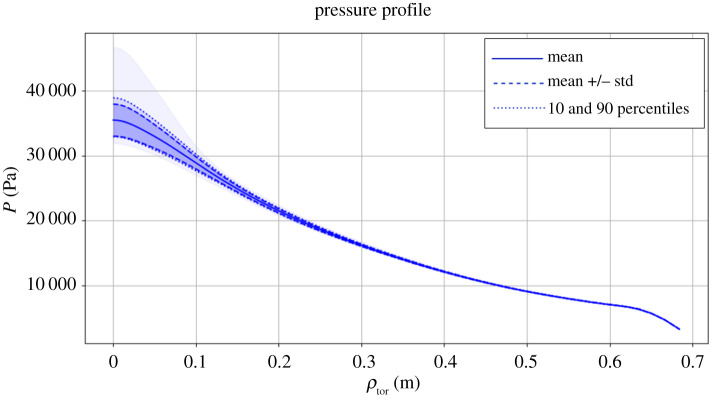
Descriptive statistics and complete range of the pressure *P*. (Online version in colour.)

The descriptive statistics as well as the complete range of the output pressure *P* with respect to the radial coordinate *ρ*_tor_ are shown in figure 6, and they correspond to the expected values [21,24]. The results between UQP1 and UQP2 are qualitatively the same: the differences between output means of each UQP are in the order of $O(10^{-5})$. The main advantage in this case study is the flexibility given by the usage of UQP2, where we can easily perform a size reduction for an expensive code without affecting the other submodels. In this case, this improved performance by almost a factor of 2 compared to the UQP1 with PCE, which is a fairly good result since the most expensive model (the equilibrium) needs 3 uncertain parameters *C*_1_, *C*_3_ and *C*_4_ (*C*_2_ = *C*_1_ to force the derivative to be zero on *ρ*_tor_ = 0) instead of the initial six parameters.

Note that MUSCLE 3 was not applied in this example since the work was done to prove the concept of UQP2 before the development of MUSCLE 3. A fairly large amount of effort has been spent on wiring up submodels and resetting inputs/outputs. This also implies the importance and convenience of applying MUSCLE 3 in UQP2 or UQP3 cases in which the structure (resampling/coordination in between the submodels) or the component (replacing the original submodel with a surrogate model) of a multiscale model might be changed.

The example demonstrated above is the first step towards our final goal: the UQ for the complete fusion model, in which two more submodels are involved—a full 3D gyrofluid model that determines the fluxes, and a module that converts these fluxes to transport coefficients (required inputs to Transport model). Contrary to the example above, the complete fusion model is a cyclic model. In the future, we plan to apply UQP3 to the complete cyclic plasma fusion model with the support of MUSCLE 3.

### (b) Case study two: towards a cyclic multiscale application

In this section, we will give a demonstration towards UQP3-B, i.e. a cyclic multiscale model with surrogate optimization. Specifically, we will focus on the two-dimensional Gray–Scott reaction–diffusion model [25], in which two chemical species *U* and *V* react according to *U* + 2*V* → 3*V* and *V* → *P*. The system is modelled by a set of partial differential equations for the local concentrations of *U* and *V*, denoted by *u*(*x*, *y*, *t*) ∈ [0, 1] and *v*(*x*, *y*, *t*) ∈ [0, 1]. The quantities of *u* and *v* are non-dimensional, and the values of 0 and 1 represent the minimum and maximum local concentrations respectively. We introduce the decomposition $u=\bar{u}+u^{\prime }$ and $v=\bar{v}+v^{\prime }$, where $\bar{u}$ and $\bar{v}$ represent the macroscopic concentrations, defined as the part of *u* and *v* which we are able to resolve on a spatial grid of 128 × 128 nodes. Conversely, *u*′ and *v*′ represent the unresolved microscale spatial components. The macroscopic governing equations are as follows:

6.2 $$\frac{\partial \bar{u}}{\partial t}=D_{u}\nabla^{2}\bar{u}-\bar{u}{\bar{v}}^{2}+f(1-\bar{u})+\bar{G_{u}(u,v)},\;\frac{\partial \bar{v}}{\partial t}=D_{v}\nabla^{2}\bar{v}+\bar{u}{\bar{v}}^{2}-(f+k)\bar{v}+\bar{G_{v}(u,v)}.$$

Chemical *U* is added to the system at a feed rate given by the model constant *f*, and *V* is removed at a ‘kill’ rate *f* + *k*, where *k* is another model constant. We specify diffusion coefficients *D*_*u*_ = 2 × 10^−5^ and *D*_*v*_ = 10^−5^, and use a 2.5 × 2.5 spatial domain with periodic boundary conditions. The nonlinear reaction term *R*(*u*, *v*): = *uv*^2^ does not commute with the projection operator, i.e. $\bar{R(u,v)}\neq R(\bar{u},\bar{v})$, which gives rise to two additional unclosed subgrid-scale terms *G*_*u*_ and *G*_*v*_. Hence, the microscopic scales arise due to the part of the nonlinear reaction term that cannot be resolved on our macroscopic grid, i.e. $\bar{uv^{2}}-\bar{u}{\bar{v}}^{2}$.

Our aim here is to close the system by replacing $\bar{G_{u}}$ and $\bar{G_{v}}$ with data-driven surrogate models. We generate a database of training data by solving the Gray–Scott equations for *u* and *v* at a high spatial resolution of 512 × 512 nodes. Instead of creating a surrogate for the spatially dependent subgrid-scale terms, we create the so-called reduced surrogates [26]. These are specifically geared towards predicting global (i.e. spatially integrated) QoIs of the form

6.3 $$Q_{i}(t)=\frac{1}{A}\int \int q_{i}(\bar{u},\bar{v};x,y,t)\;\text{d}x\text{d}y.$$

Here, *q*_*i*_ is some function of the primitive variables and *A* is the area of the spatial domain. For instance, let us define our QoIs by the following set of integrands: $\left\{q_{1}=\bar{u},q_{2}={\bar{u}}^{2},q_{3}=\bar{v},q_{4}={\bar{v}}^{2}\right\}$, i.e. we are interested in the average concentration of *U* and *V*, as well as the average squared concentrations. The task of the reduced subgrid-scale surrogates is to ‘track’ the reference QoIs, i.e. to keep $Q_{i}^{\text{ref}}(t)-Q_{i}(t)$ small for all times during training, where $Q_{i}^{\text{ref}}$ is the reference QoI computed from the high-resolution training data. We skip details for the sake of brevity, and refer to [26] for more technical information, or to [27] for the code and a practical tutorial.

**Figure 7.**
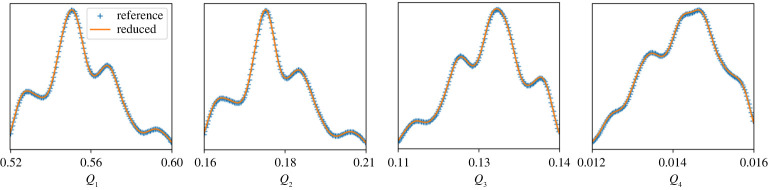
Probability density functions (PDFs) of *Q*_1_ to *Q*_4_ for both the high-resolution reference model and the low-resolution model with reduced subgrid-scale term. The PDFs are computed using data from 50000 time steps. (Online version in colour.)

The results for the training phase of the reduced surrogate are shown in figure 7. This shows the PDFs of the four QoIs, for both the high spatial resolution simulation (with no subgrid-scale terms), and the low-resolution model (6.2) with reduced surrogates. As this is the training phase, these reduced surrogates are informed directly from the training data [26]. Note that the two PDFs for each *Q*_*i*_ are practically indistinguishable from each other, confirming the accuracy of the reduced surrogate during the training phase.

The high-resolution simulation had a wall-clock time of roughly 223 min (for 50 k time steps), whereas the reduced model completed in 24 min, which is more than nine times faster. Using a 1024 × 1024 spatial resolution for the reference model yielded a wall-clock time of roughly 19 h. The reduced model, still at 24 min, is therefore 48 times faster. For problems in three spatial dimensions, the speed-up will increase further.

That said, it is important to point out that these speed-ups, denoted by *S*, compare the cost of running the expensive (reference) model $C_{\mu }$ with the cost of the (reduced) surrogate $C_{\mu }^{*}$, i.e. $S:=C_{\mu }/C_{\mu }^{*}$. The cost of sampling the reference model in the first place to create $C_{\mu }^{*}$, denoted by *C*_*k*_ in (5.11), is not yet included. Let us assume that each sample *n* = 1, …*N* of the inputs (*f* and *k* in our case, see below) will require the construction of its own surrogate. As noted, a data-driven surrogate model will require sampling from $C_{\mu }$, which, generally speaking, we can do for $N_{\Delta t_{M}}/T$ time steps, where *T* ≥ 1. The cost of sampling the reference model is therefore given by $C_{k}=N_{\Delta t_{M}}NC_{\mu }/T$. We can now write (5.11) as

6.4 $$\frac{C_{{\text{UQP1}}_{\mu }}}{C_{{\text{UQP3-B}}_{\mu }}}=\frac{N_{\Delta {\text{t}}_{M}}NC_{\mu }}{N_{\Delta {\text{t}}_{M}}NC_{\mu }^{*}+C_{k}}=\frac{N_{\Delta {\text{t}}_{M}}NC_{\mu }}{N_{\Delta {\text{t}}_{M}}NC_{\mu }/S+N_{\Delta t_{M}}NC_{\mu }/T}=\frac{ST}{S+T}.$$

Thus the final speed-up is given by *ST*/(*S* + *T*), where *S* is the speed-up obtained by replacing $C_{\mu }$ with $C_{\mu }^{*}$, and *T* ≥ 1 reflects how much we had to sample $C_{\mu }$ in order to construct $C_{\mu }^{*}$. A higher value of *T* means less $C_{\mu }$ training data is required to construct $C_{\mu }^{*}$. Note that lim _*S*→∞_*ST*/(*S* + *T*) = *T*, which gives the maximum speed-up which can be obtained for UQP3-B with a data-driven surrogate. If for instance $C_{\mu }$ must be executed for 50% of the $N_{\Delta t_{M}}$ time step in order to get an accurate $C_{\mu }^{*}$, (6.4) is limited to ≤2. If only 1% is sufficient, the speed-up is bounded by 100. Finally, we mention that *S* controls how fast (6.4) approaches the limit with increasing *T*.

We have demonstrated the capability of creating surrogates with *S* ≫ 1, and have provided theoretical bounds on the final speedup. Extrapolating the reduced surrogate beyond the training phase (*T* > 1) is the subject of ongoing research. In the remainder, we therefore set *T* = 1, such that the final UQ results (figure 9) are virtually exact, although we cannot report a final speed-up yet. Note that in earlier work on the Gray–Scott model, which focused on time-scale separation, we were able to accelerate UQ using surrogates [14].

**Figure 8.**
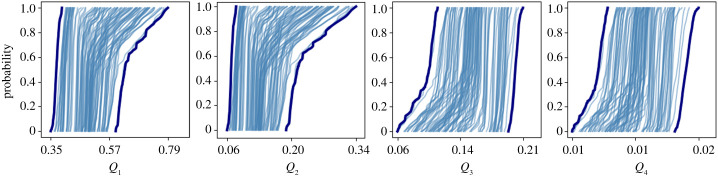
Probability boxes, with *Q*_*i*_ on the horizontal axis, and probability on the vertical. Thin lines show individual ECDFs computed from *Q*_*i*_ time-series data for a fixed value of *f* and *k*. Thick lines represent the probability box formed by taking the envelope of all ECDFs.

**Figure 9.**
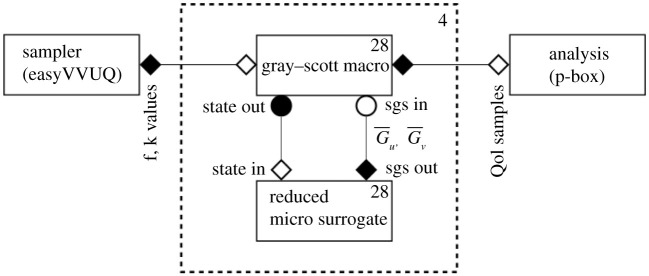
The MUSCLE 3 diagram for this particular implementation of UQP3-B. The micro model has been replaced by a reduced surrogate, which receives the macroscopic state, and returns the ${\bar{G}}_{u}$ and ${\bar{G}}_{v}$ subgrid-scale (sgs) terms. The EasyVVUQ sampler provides the randomly drawn values of *f* and *k* to the macro model, and the MUSCLE 3 manager creates 28 different copies of the coupled system. Finally, this process is repeated on four different nodes of the Eagle supercomputer, leading to 112 output samples from which we compute the probability boxes.

The Gray–Scott system is very sensitive to *f* and *k*, see [25]. We prescribe f∼U[0.02,0.025] and k∼U[0.05,0.055], designed to explore a region that generates time-dependent spiral or spotted structures, depending on the value of *f* and *k*. We coupled the macroscopic Gray–Scott equations (6.2) to a reduced surrogate (stored in a separate Python script), using MUSCLE 3, see figure 8. The sampler we used to draw the samples from the *f* and *k* distributions was EasyVVUQ [22], and we computed the ensemble in parallel on the Eagle supercomputer at the Poznan Supercomputing and Networking Center. The MUSCLE 3 manager runs on a single node, and was configured to spawn 28 copies of the coupled system with different *f* and *k* values, as one node contains 28 cores on Eagle. As we submitted to 4 nodes simultaneously, our ensemble therefore consists of 4 × 28 = 112 samples. Our output statistics of interest are the probability boxes of *Q*_1_ to *Q*_4_. These are defined as the envelope formed by the 100 empirical cumulative distribution functions (ECDFs), computed from the *Q*_*i*_ time series, see figure 9. The spread of the different ECDFs is significant, showing (as expected), substantial uncertainty due to imperfectly known values of *f* and *k*.

More examples of UQP3-A and UQP3-B can be found in [14,28,29] where the UQP3-A and UQP3-B were applied to a different reaction–diffusion model and a 2D multiscale in-stent restenosis model, and achieved a significant speed-up of UQ. We invite the readers to these papers for further details.

## 7. Conclusion and future work

In this paper, we described a number of patterns for efficient UQ of multiscale models, and categorized them by the level of intrusiveness and optimization method. These UQPs provide the basic building blocks to create tailored UQ for multiscale models. We showed how these methods can be implemented in multiscale models using the formalism of the MMSF and the MUSCLE 3 coupling toolkit. Two case studies were presented to show the application of the patterns.

We have discussed UQP implementation for forward UQ and SA. The implementation of UQPs for inverse UQ analysis will be explored in the future. The implementation will require generating samples based on the previous results, e.g. using the Markov-chain Monte Carlo method.

## Data accessibility

This article has no additional data.
